# Supplementary material for: Solution-Mediated Inversion of SnSe to Sb2Se3 Thin-Films
Source: Nanomaterials (Basel). 2022 Aug 23;12(17):2898. doi: 10.3390/nano12172898 (PMC9458253; doi:10.3390/nano12172898)
Supplement: Supplementary file 1 [file nanomaterials-12-02898-s001.zip › nanomaterials-1855473-supplementary.pdf]

Solution-Mediated Inversion of SnSe to Sb_2Se_3 Thin-Films

Svetlana Polivtseva [1,*], Julia Kois [2], Tatiana Kruzhilina [1], Reelika Kaupmees [1], Mihhail Klopov [3], Palanivel Molaiyan [4], Heleen van Gog [5], Marijn A. van Huis [6] and Olga Volobujeva [1]

[1] Department of Materials and Environmental Technology, School of Engineering, , TalTech, Ehitajate tee 5, 19086 Tallinn, Estonia
[2] Auramet Solutions OÜ, Kalliomäentie 1B, 02920 Espoo, Finland
[3] Department of Cybernetics, School of Science, TalTech, Ehitajate tee 5, 19086 Tallinn, Estonia
[4] Research Unit of Sustainable Chemistry, Faculty of Technology, University of Oulu, Pentti Kaiteran katu 1, 90014 Oulu, Finland
[5] Nanostructured Materials and Interfaces, Zernike Institute for Advanced Materials, University of Groningen, Nijenborgh 4, 9747 AG Groningen, The Netherlands
[6] Soft Condensed Matter, Debye Institute for Nanomaterials Science, Utrecht University, Princetonplein 5, 3584 CC Utrecht, The Netherlands
* Correspondence: cvpolcv@gmail.com

Table S1 Redox equilibria considered and dominant regions of solute species in the Sb_2S_3–H_2O and Sb_2Se_3–H_2O systems.

**Redox equilibria**	**Reaction number**
$2SbO^+ + 3S + 4H^+ + 6e^- \rightleftarrows Sb_2S_3 + 2H_2O$	**1**
$2HSbO_{2(aq)} + 3S + 2H^+ + 6e^- \rightleftarrows Sb_2S_3 + 4H_2O$	**2**
$2Sb(OH)_4^- + 3S + 8H^+ + 10e^- \rightleftarrows Sb_2S_3 + 8H_2O$	**3**
$2Sb(OH)_6^- + 3S + 12H^+ + 6e^- \rightleftarrows Sb_2S_3 + 12H_2O$	**4**
$Sb_2S_3 + 6H^+ + 6e^- \rightleftarrows 2Sb + 3H_2S$	**5**
$Sb_2S_3 + 3H^+ + 6e^- \rightleftarrows 2Sb + 3HS^-$	**6**
$Sb_2S_3 + 6e^- \rightleftarrows 2Sb + 3S^{2-}$	**-**
$Sb(OH)_6^- + 4H^+ + 2e^- \rightleftarrows SbO^+ + 5H_2O$	**7**
$Sb(OH)_6^- + 3H^+ + 2e^- \rightleftarrows HSbO_{2(aq)} + 4H_2O$	**8**
$Sb(OH)_6^- + 2H^+ + 2e^- \rightleftarrows Sb(OH)_4^- + 2H_2O$	**9**
$SbO^+ + 2H_2O \rightleftarrows HSbO_{2(aq)} + H^+$	**-**
$HSbO_{2(aq)} \rightleftarrows Sb(OH)_4^- + 3H^+$	**-**
$Sb_2S_3 + HS^- \rightleftarrows Sb_2S_4^{2-} + H^+$	**10**
H_2S / HS^-	**-**
HS^- / S^{2-}	**-**
H_2SO_4 / HSO_4^-	**-**
HSO_4^- / SO_4^{2-}	**-**
$2SbO^+ + 3Se + 4H^+ + 6e^- \rightleftarrows Sb_2Se_3 + 2H_2O$	**1***
$2HSbO_{2(aq)} + 3Se + 2H^+ + 6e^- \rightleftarrows Sb_2Se_3 + 4H_2O$	**2***
$2Sb(OH)_4^- + 3Se + 8H^+ + 10e^- \rightleftarrows Sb_2Se_3 + 8H_2O$	**3***
$2Sb(OH)_6^- + 3Se + 12H^+ + 6e^- \rightleftarrows Sb_2Se_3 + 12H_2O$	**-**
$Sb_2Se_3 + 6H^+ + 6e^- \rightleftarrows 2Sb + 3H_2Se$	**4***

$Sb_2Se_3 + 3H^+ + 6e^- \rightleftarrows 2Sb + 3HSe^-$	**5***
$Sb_2Se_3 + 6e^- \rightleftarrows 2Sb + 3Se^{2-}$	**6***
$Se + 3H_2O \rightleftarrows H_2SeO_3 + 4H^+ + 4e^-$	**-**
$Se + 3H_2O \rightleftarrows HSeO_3^- + 5H^+ + 4e^-$	**7***
$Se + 3H_2O \rightleftarrows HSeO_3^{2-} + 6H^+ + 4e^-$	**8***
H_2SeO_3 / $HSeO_3^-$	**-**
$HSeO_3^-$ / $HSeO_3^{2-}$	**-**
H_2Se / HSe^-	**-**
HSe^- / Se^{2-}	**-**
$H_2 \rightleftarrows 2H^+ + 2e^-$	**a**
$2H_2O \rightleftarrows O_2 + 4H^+ + 4e^-$	**b**

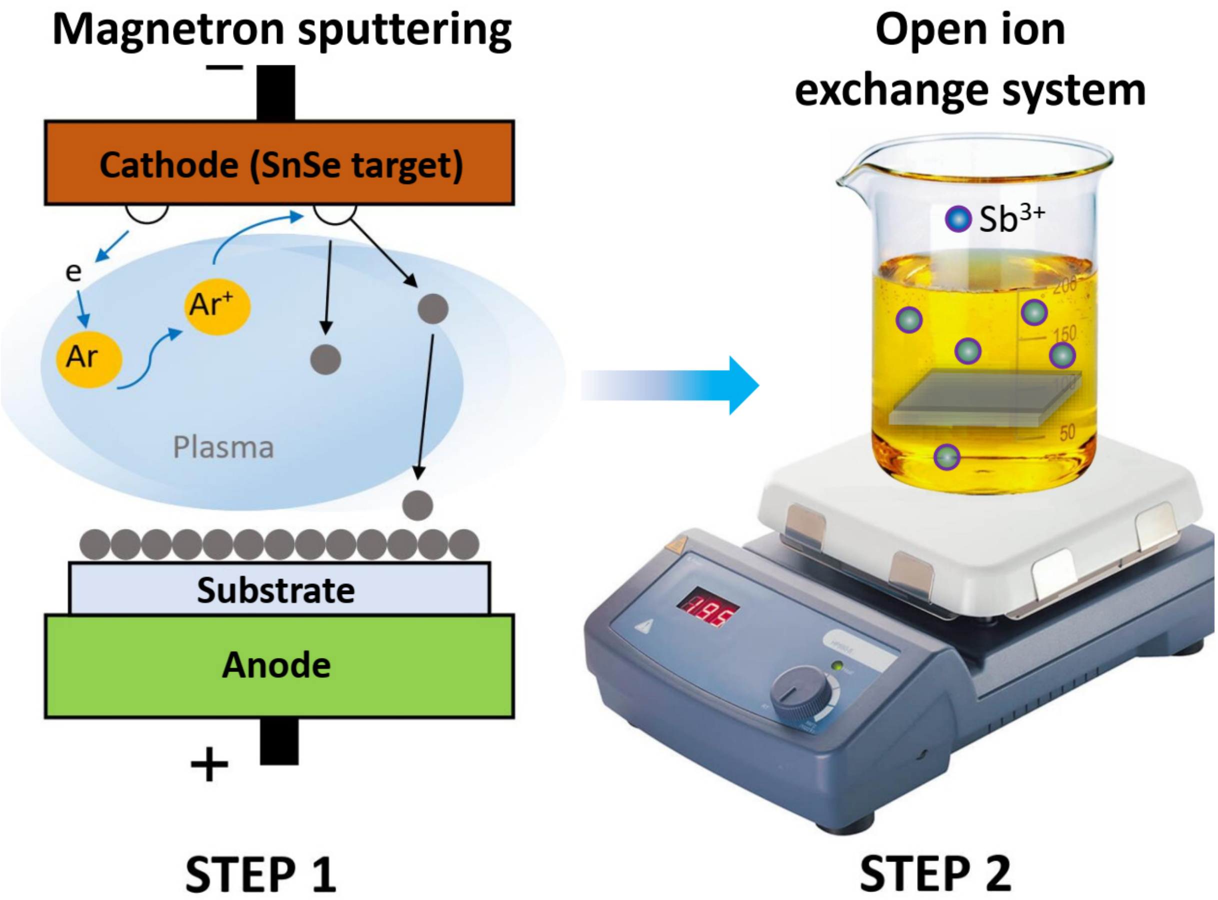

Figure S1: A sketch for the fabrication of Mo/IE-Sb_2Se_3 structures using a combinative approach of magnetron sputtering and cation exchange.

Effect of $SbCl_3$ concentration

Table S2 Atomic percentages of tin, antimony, and selenium according to the EDX data, main phase according to the XRD and Raman data, the average crystallite size (D), and lattice parameters of the main phase developed within 22 min depending on the molarity (M) of $SbCl_3$ in glycerol. Each parameter was determined by averaging the values obtained from three measurements for each sample. The error represents the standard deviation.

Treatment	M (mM)	elements (at. %)			main phase	D (nm) (± 1)	Lattice parameters (Å) (± 0.001)		
		Sn	Se	Sb			*a*	*b*	*c*
pristine		51.0	49.0	-	SnSe	20	11.670	4.201	4.353
$SbCl_3$-glycerol	**11**	42.0	50.5	7.5	SnSe	31	11.468	4.198	4.353
	**22**	-	60.1	39.9	**Sb_2Se_3**	75	11.597	11.740	3.970
	**33**	-	60.0	40.0	**Sb_2Se_3**	83	11.610	11.724	3.970
	**44**	-	60.0	40.0	**Sb_2Se_3**	97	11.614	11.719	3.970
Reference	PDF Card No.: 01-089-0821				Sb_2Se_3		11.588	11.744	3.955

Table S3 Deconvoluted Raman peak positions of the pristine SnSe film deposited on Mo-coated substrates and those treated in 11, 22, 33, and 44 mM $SbCl_3$ solutions for 22 min at ~210 °C. The error represents the standard deviation.

Main phase	M (mM)	Raman shift (cm^{-1}) (± 0.3)							
		Peak 1	Peak 2	Peak 3	Peak 4	Peak 5	Peak 6	Peak 7	Peak 8
SnSe	bare	70.4	97.1	119.5		156.0	184.0		
	**11**	69.9	95.3	118.4		155.0	180.7		
Sb_2Se_3	**22**		98.7	116.0	129.0	153.3		190.8	211.9
	**33**		98.4	116.2	128.9	153.5		191.0	212.0
	**44**		98.9	116.4	129.0	153.4		191.1	211.9

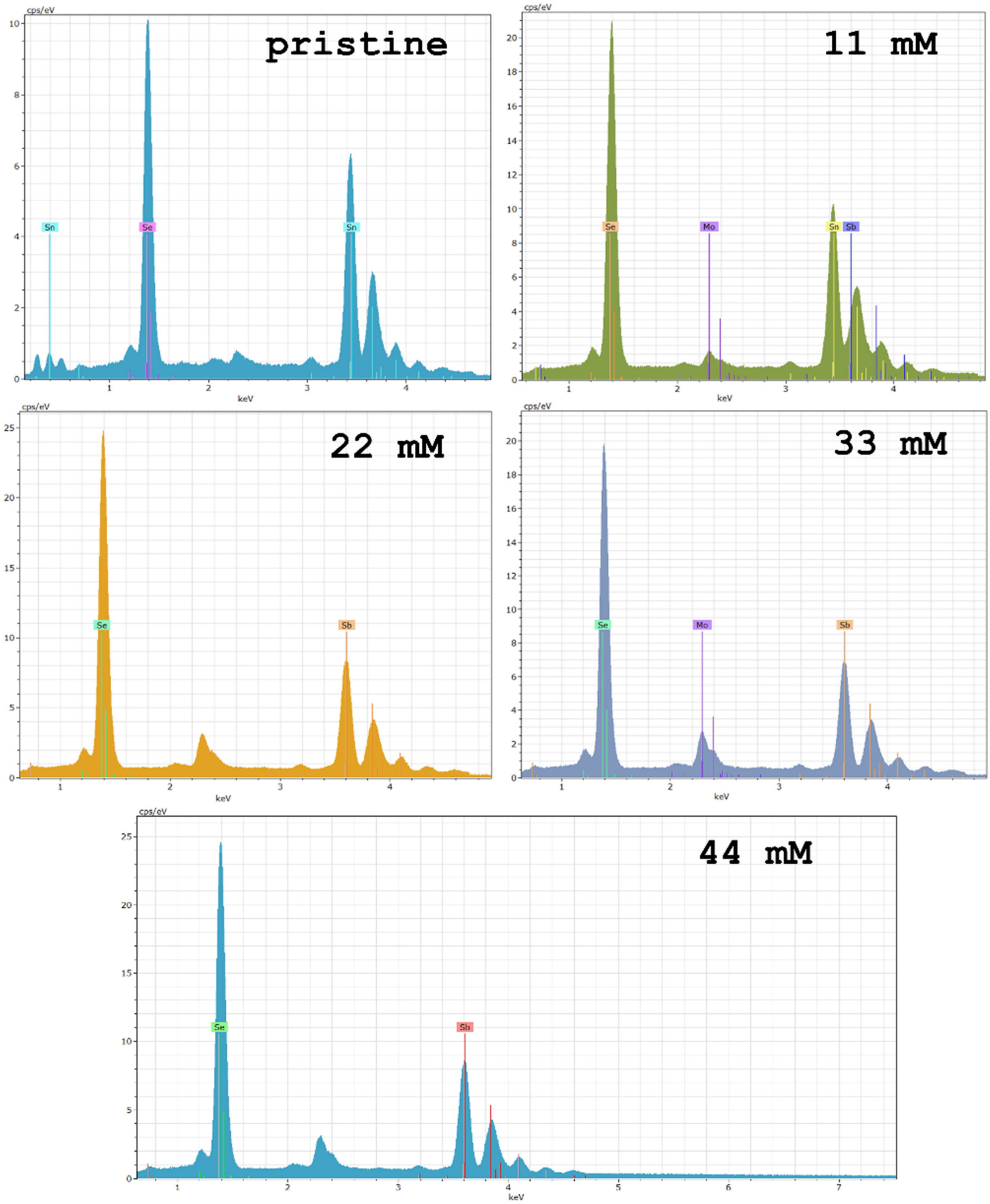

Figure S2. EDX spectra of the pristine SnSe film and those treated in glycerol at various concentrations of $SbCl_3$ for 22 min at ~210 °C.

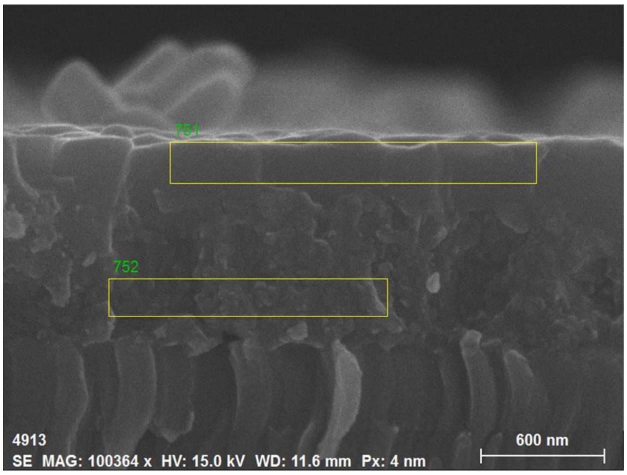

Spectrum: 751

Element	Series	unn. C [wt.%]	norm. C [wt.%]	Atom. C [at.%]	Error (1 Sigma) [wt.%]
Tin	L-series	37.22	42.60	35.53	1.15
Antimony	L-series	14.87	17.02	13.84	0.48
Selenium	L-series	35.27	40.37	50.62	1.76
	Total:	87.36	100.00	100.00	

Spectrum: 752

Element	Series	unn. C [wt.%]	norm. C [wt.%]	Atom. C [at.%]	Error (1 Sigma) [wt.%]
Tin	L-series	47.31	55.13	45.99	1.14
Antimony	L-series	3.26	4.88	3.85	0.28
Selenium	L-series	34.36	39.99	50.16	1.52
	Total:	84.93	100.00	100.00	

Figure S3. Cross-sectional EDX analysis of the distribution of Sn, Sb, and Se elements composing the surface (751) and bottom (752) parts of the film treated in 11 mM $SbCl_3$ solution for 22 min.

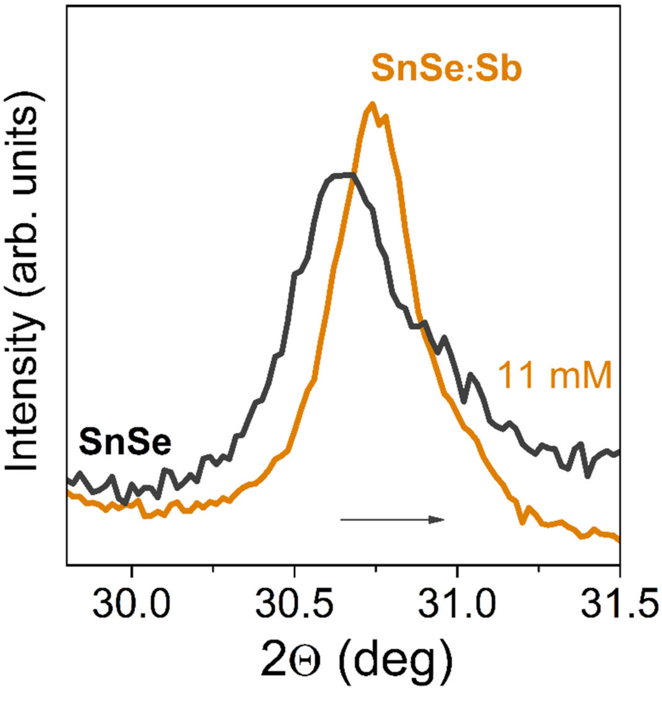

Figure S4. Displacement of the (111) XRD peak of the pristine SnSe film deposited on Mo-coated substrates and those treated in 11 mM $SbCl_3$ for 22 min at ~210 °C.

X-ray photoelectron spectroscopy (XPS)

XPS analysis was performed using a Kratos Axis Ultra DLD X-ray photoelectron spectrometer equipped with achromatic dual anode Mg Kα (1253.6 eV)/Al Kα (1486.6 eV) and monochromatic Al Kα (1486.6 eV) X-ray sources. Spectra were calibrated assuming the C 1s peak at 284.6 eV. Ag^+ ion source was used with 4 keV (0.1 mA cm^{-2}, 60 s) to eliminate atmospheric contaminants from the surface.

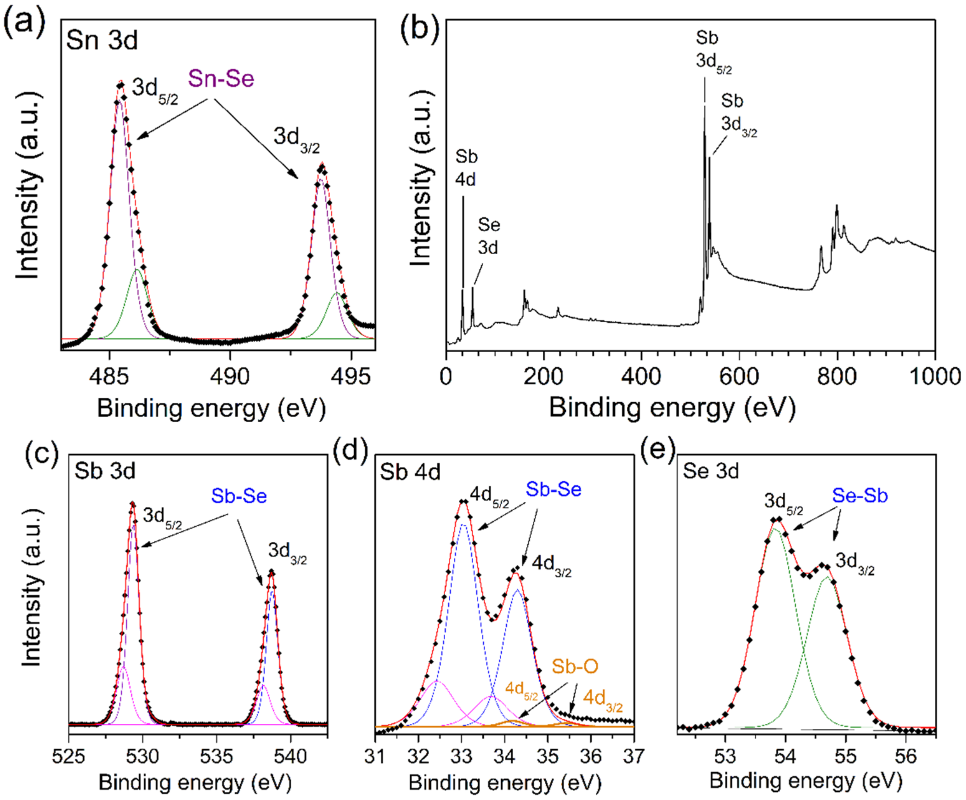

Figure S5. X-ray photoelectron spectroscopy showing the Sn 3*d* (**a**) core level peak region of the pristine SnSe film; (**b**) XPS survey spectrum of the Sb_2Se_3 layer experienced cation exchange in 44 mM $SbCl_3$ solutions for 22 min; high-resolution spectra of Sb 3*d* (**c**) and Sb 4*d* (**d**), and Se 3*d* (**e**) core level peak regions.

Pristine SnSe XPS characterization reveals the Sn 3*d* peaks at 485.5 eV (Sn $3d_{5/2}$) and 493.8 eV (Sn $3d_{3/2}$), indicative of the SnSe binding energies (Fig. S5). The binding energies of the components are grouped in Table S4. The peak-differentiation-imitating analysis indicates a small amount of Sn^{4+} represented by the distinctive peaks at 486.1 and 494.4 eV.

Table S4 Measured binding energy for different states of elements

**Layer**	XPS Peak	Binding energy (eV)
SnSe	Sn(Se) $3d_{5/2}$	485.5
	Sn(Se) $3d_{3/2}$	493.8
Sb_2Se_3	Sb(Se) $3d_{5/2}$	529.4
	Sb(Se) $3d_{3/2}$	538.7
	Sb $3d_{5/2}$	528.7
	Sb $3d_{3/2}$	538.2
	Sb(Se) $4d_{5/2}$	33.0
	Sb(Se) $4d_{3/2}$	34.3
	Sb(O) $4d_{5/2}$	34.2
	Sb(O) $4d_{3/2}$	35.4
	Sb $4d_{5/2}$	32.4
	Sb $4d_{3/2}$	33.7
	Se(Sb) $3d_{5/2}$	53.8
	Se(Sb) $3d_{3/2}$	54.7
	Se $3d_{5/2}$	49.9
	Se $3d_{3/2}$	55.7
	C 1s	284.8
	O 1s	530.7

Ion exchanged Sb_2Se_3 XPS studies were further conducted to evaluate the composition of Sb_2Se_3 after completing cation exchange. It is noteworthy that the absence of carbon atoms (C 1*s* at 284.8 eV) after Ar^+ ion etching for 60 s indicates a low ability of glycerin to contaminate the formed Sb_2Se_3 layer. XPS analysis highlights the disappearance of Sn 3*d* peaks at 485.5 eV (Sn $3d_{5/2}$) and 493.8 eV (Sn $3d_{3/2}$) after the complete inversion of SnSe in Sb_2Se_3, which is consistent with XRD, Raman, and EDX data presented in the main text. Panels c, d, and e in Figure 5S present magnified XPS spectra of Sb 3*d,* Sb 4*d,* and Se 3*d* core levels after Ar^+ ion etching for 60 s (~1 nm thickness). In the Sb 3*d* region, XPS reveals a small degree of Sb_2Se_3 splitting as indicated by the distinctive peaks at 528.7 eV (Sb $3d_{5/2}$) and 538.2 eV (Sb $3d_{3/2}$), characteristics of metallic Sb binding energies (Table S4). The formation of metallic species because of argon etching is expected for metastable compounds. Insignificant oxidation of Sb_2Se_3 can be assumed from the deconvoluted Sb 4*d* spectrum comprising barely noticeable peaks at 34.2 eV (Sb $4d_{5/2}$) and 35.4 eV (Sb $4d_{3/2}$), the binding energies of Sb_2O_3. A low level of oxide contamination in the near-surface layer meets our expectations considering fabricating techniques used in this work. In the Se 3*d* region, the binding energies of the Se component at 53.8 eV (Se $3d_{5/2}$) and 54.7 eV (Se $3d_{3/2}$) associated with Sb_2Se_3 are in good agreement with the expected values. Peak deconvolution using the Gaussian-Lorentzian function shows no contributions from elemental Se. Thus, the bulk of our sample inverted by cation exchange consists of pure Sb_2Se_3 with some residual Sb_2O_3 in the near-surface layer and elemental Sb formed upon Ar^+ ion etching.

Effect of time treatment

Table S5 Atomic percentages of tin, antimony, and selenium according to the EDX data, the main phase according to XRD and Raman, the average crystallite size (D), and lattice parameters developed in 44 mM $SbCl_3$ solutions within different time treatments. Each parameter was determined by averaging the values obtained from three measurements for each sample. The error represents the standard deviation.

Treatment	time (min)	elements (at. %)			*main phase*	D (nm) (± 1)	Lattice parameters (Å) (± 0.001)		
		Sn	Se	Sb			*A*	*b*	*C*
pristine	bare	51.0	49.0	-		20	11.670	4.201	4.353
44 mM $SbCl_3$	**5**	51.0	49.0	-	SnSe	40	11.611	4.216	4.397
	**6**	51.0	49.0	-	SnSe	25	11.572	4.206	4.383
	**7**	50.2	48.5	1.3	SnSe	22	11.534	4.204	4.440
	**8**	50.1	48.4	1.5	SnSe	21	11.505	4.202	4.343
	**9**	43.5	49.9	6.6	SnSe	20	11.441	4.185	4.403
	**10**	42.7	49.4	7.9	SnSe	19	11.420	4.163	4.441
	**12**	37.0	51.6	11.4	*Sn_3SbSe_4**				
	**17**	-	60.1	39.9	**Sb_2Se_3**	79	11.611	11.737	3.970
	**22**	-	60.0	40.0	**Sb_2Se_3**	97	11.611	11.719	3.970
Reference	PDF Card No.: 01-089-0238				SnSe		11.611	4.216	4.397

*Proposed ternary tin-antimony selenide phase formed in this work.

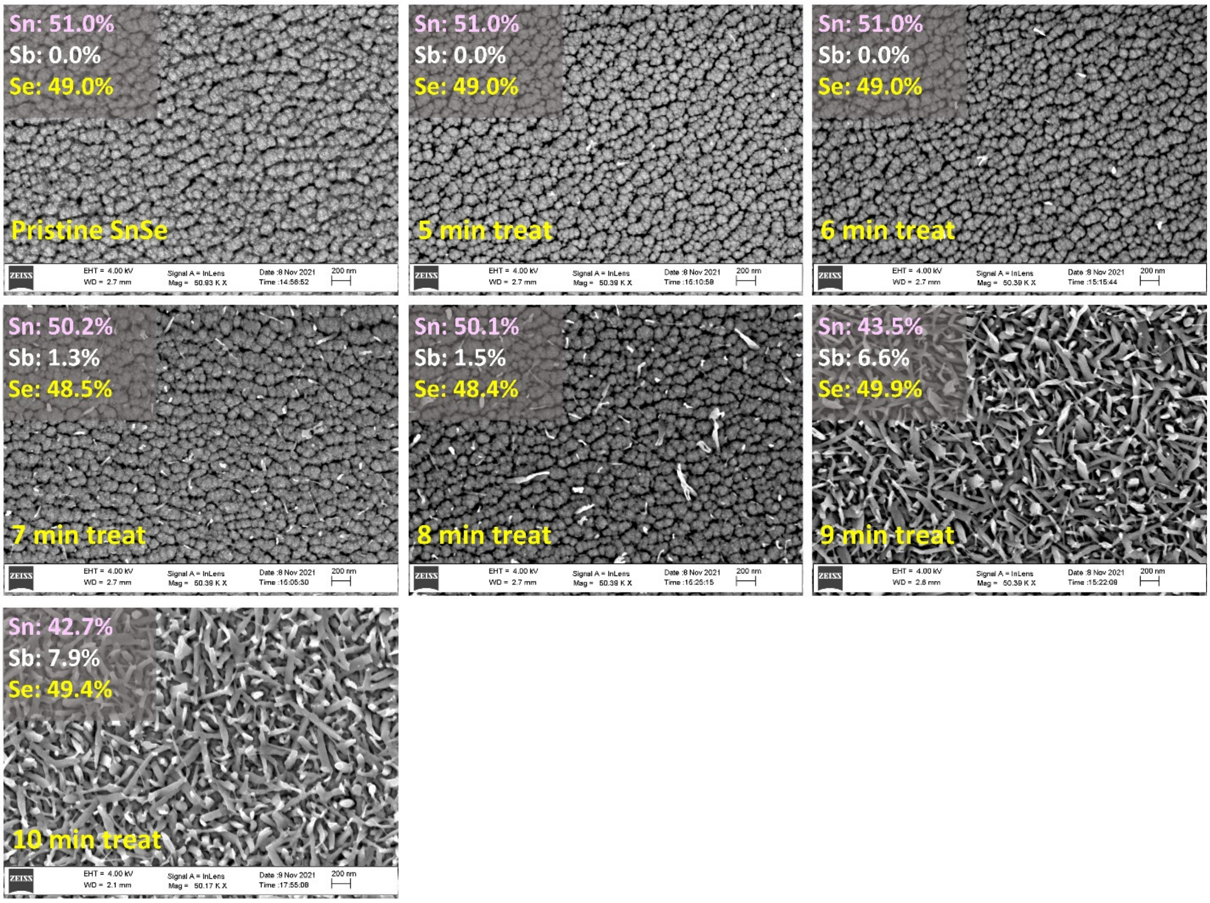

Figure S6. SEM images presenting the morphology evolution of SnSe thin films treated in 44 mM $SbCl_3$ solution for a different time.

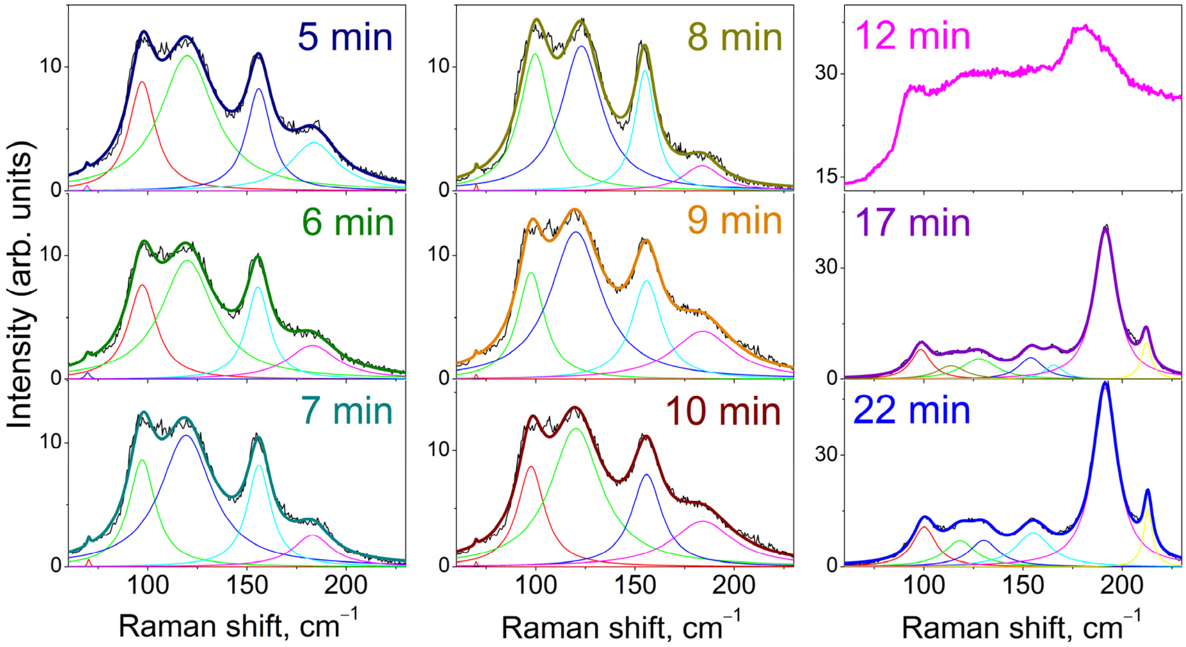

Figure S7. Raman spectra of films treated in 44 mM $SbCl_3$ solutions for a different time.

Table S6 Deconvoluted Raman peak positions of the pristine SnSe film deposited on Mo-coated substrates and those treated in 44 mM $SbCl_3$ solutions for a different time at ~210 °C. The error represents the standard deviation.

Main phase	time (min)	Raman shift (cm^{-1}) (± 0.3)							
		Peak 1	Peak 2	Peak 3	Peak 4	Peak 5	Peak 6	Peak 7	Peak 8
SnSe	bare	70.4	97.1	119.5		156.0	184.0		
	**5**	70.4	97.1	119.5		156.0	184.0		
	**6**	70.2	97.1	119.4		155.5	183.0		
	**7**	70.2	97.1	119.2		155.5	182.9		
	**8**	70.1	99.5	123.0		155.0	182.8		
	**9**	70.1	97.7	121.0		155.8	184.1		
	**10**								
Sb_2Se_3	**17**		98.5	113.7	127.8	153.8		191.0	212.1
	**22**		100.1	116.2	130.2	155.1		191.3	212.9

Chemical reactions in multi-ion systems initiating cation exchange

$SbCl_3 + HOCH_2\text{-}CH(OH)\text{-}CH_2OH \rightleftarrows Sb(C_3O_3H_4) + 3HCl$ (Eq. S1)

$Sn_{(1+x)}Se + H^+ \rightarrow SnSeSn{:}H + Cl^- \rightarrow SnSeSnCl + 1/2H_2$ (Eq. S2)

$(SnSe_b)(Sn_2)_{GB} + 2Cl^- = (SnSe_b)((SnCl)_2)_{GB}$ (Eq. S3)

$(SnSe_b)((SnCl)_2)_{GB} = (SnSe_b)(SnV_{Sn})_{GB} + SnCl_2$ (Eq. S4)

$(SnSe_b)(SnV_{Sn})_{GB} + Sb^{3+} + Cl^- = (SnSe_b)(SnSbCl)_{GB}$ (Eq. S5)

$(SnSe_b)((SnCl)_2)_{GB} + SbCl_3 = (SnSe_b)V_{Sn}SbCl_{GB} + 2SnCl_2$ (Eq. S6)

Overall reaction

$3SnSe_{(s)} + 2SbCl_{3(sol)} \rightarrow Sb_2Se_{3(s)} + 3SnCl_{2(sol)}$ (Eq. S7)

Table S7. Overview of DFT-calculated phases. Symmetry groups, *k*-mesh used, number of atoms in the conventional unit cell or considered supercell, calculated lattice parameters, and Bader charges.

Phase	Space group (nr).	atoms per cell	k-mesh	Lattice parameters	Bader charges (e)
**Reference phases**					
Sn (beta)	$I4_1$/amd (141)	4	20×20×36	a=5.943 Å<br>c=3.217 Å	Sn: 0.00
Sb	R-3m (166)	6	28×28×10	a=4.378 Å<br>c=11.498 Å	Sb: 0.00
Se	$P3_121$ (152)	3	10×10×10	a=4.513 Å<br>c=5.051 Å	Se: 0.00
SnSb	Fm-3m (225)	8	20×20×20	a=6.188 Å	Sb: +0.14<br>Sn: –0.14
SnSe	Pnma (62)	8	10×10×4	a=4.205 Å<br>b=4.569 Å<br>c=11.783 Å	Sn: +0.76<br>Se: –0.76
$SnSe_2$	P-3m1 (164)	3	18×18×10	a=3.873 Å<br>c=6.924 Å	Sn: +0.84<br>Se: –0.42
Sb_2Se_3	Pnma (62)	20	12×4×4	a=4.029 Å<br>b=11.535 Å<br>c=12.835 Å	Sb: +0.74<br>Se: –0.44, –0.51, –0.53
Cl_2	molecule	2	4×4×4	N.A.	Cl: 0.00
$SnCl_2$	Pnma (62)	12	10×6×6	a=4.309 Å<br>b=8.111 Å<br>c=10.340 Å	Sn: +1.16<br>Cl: –0.58
**Supercells**					
$Sn_{1-x}Sb_xSe$ (Fig. S7)	2×2×1 super	32	6×6×4	a~8.4 Å<br>b~9.1 Å<br>c~11.8 Å	various
$Sn_{10}Sb_2Se_{12}$ (Fig. 6b)	1×3×1 super	24	10×4×4	a=4.151 Å<br>b=13.305 Å<br>c=12.072 Å<br>α=89.1°	Sn: +0.57<br>Sb: +0.64<br>Se: –0.54 to –0.59
$Sn_8Sb_4Se_{12}$ (Fig. 6c)	1×3×1 super	24	10×4×4	a=4.183 Å<br>b=14.143 Å<br>c=11.972 Å<br>α=95.3°	Sn: +0.57<br>Sb: +0.32, +0.73<br>Se: –0.51 to –0.60
$Sn_8Sb_2Se_{12}$ (Fig. 6d)	1×3×1 super	22	10×4×4	a=4.169 Å<br>b=12.879 Å<br>c=11.935 Å<br>α=87.8°	Sn: +0.64<br>Sb: +0.74<br>Se: –0.50 to –0.61
$Sn_6Sb_4Se_{12}$ (Fig. 6e)	1×3×1 super	22	10×4×4	a=4.195 Å<br>b=12.672 Å<br>c=12.883 Å<br>α=107.3°	Sn: +0.61<br>Sb: +0.71<br>Se: –0.43 to –0.62

Table S8. DFT-calculated formation enthalpies ΔH_1 and ΔH_2 were evaluated using Eqs. (3) and (4) in the main text, respectively, for pure SnSe and Sb-doped SnSe supercells. The formation enthalpies are also plotted in Figure 5.

Structure	Calculation cell	Composition	Nr. of atoms	# Sb atoms	x_{Sb}	x_{Sn}	ΔH_1 (eV/at.)	ΔH_2 (eV/at.)
pure SnSe	SnSe unit cell	Sn_4Se_4	8	0	0.000	0.500	0.000	0.000
Sb-doped	2×2×1 super	$Sn_{15}Sb_1Se_{16}$	32	1	0.031	0.469	–0.048	0.002
supercells	2×2×1 super	$Sn_{14}Sb_2Se_{16}$	32	2	0.063	0.438	–0.096	0.002
	2×2×1 super	$Sn_{13}Sb_3Se_{16}$	32	3	0.094	0.406	–0.150	–0.003
	2×2×1 super	$Sn_{12}Sb_4Se_{16}$	32	4	0.125	0.375	–0.201	–0.004
	2×2×1 super	$Sn_{14}Sb_6Se_{16}$	32	6	0.188	0.313	–0.317	–0.022
	2×2×1 super	$Sn_8Sb_8Se_{16}$	32	8	0.250	0.250	–0.432	–0.038
	2×2×1 super	$Sn_6Sb_{10}Se_{16}$	32	10	0.313	0.188	–0.544	–0.052
	2×2×1 super	$Sn_4Sb_{12}Se_{16}$	32	12	0.375	0.125	–0.659	–0.069
(Fig. 6b)	1×3×1 super	$Sn_{10}Sb_2Se_{12}$	24	2	0.083	0.417	–0.126	0.005
(Fig. 6c)	1×3×1 super	$Sn_8Sb_4Se_{12}$	24	4	0.167	0.333	–0.324	–0.061

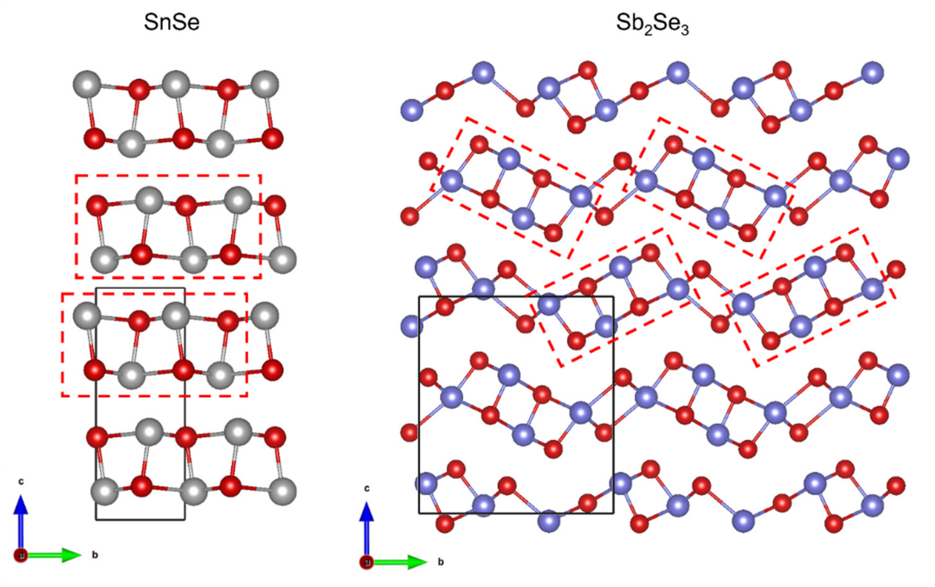

Figure S8. Crystal structures of SnSe and Sb_2Se_3 both shown in [100] projection. The black solid lines indicate the conventional unit cells. Red dashed lines indicate building blocks in Sb_2Se_3 that are isostructural to those in SnSe. Grey, blue, and red spheres denote Sn, Sb, and Se atoms, respectively.
